# Case report of conjunctival sac fistula after cosmetic lateral canthoplasty

**DOI:** 10.1186/s12886-020-01402-3

**Published:** 2020-04-03

**Authors:** Weili Zhang, Qinying Huang, Jinying Li

**Affiliations:** Ophthalmology, Peking University Shenzhen Hospital, Shenzhen, 518036 Guangdong China

**Keywords:** Conjunctival sac, Fistula, Lateral canthoplasty

## Abstract

**Background:**

To report a case of conjunctival sac fistula after cosmetic lateral canthoplasty which is rare.

**Case presentation:**

A young women who underwent bilateral canthoplasty appeared with lacrimation of the right eye. We found there was a skin fistula with transparent tears at 2 mm lateral to the right canthus ligament and the liquid containing fluorescein was seen to overflow at the fistula after using fluorescein sodium eye drops. The number 7 lacrimal duct probe was visible under the temporal conjunctiva when exploring the fistula, and the fistula was about 4 mm. The patient was diagnosed with conjunctival sac fistula and fistula excision was performed. The patient did not tear abnormally after observation 3 months later and the incision healed well.

**Conclusions:**

The case report illustrates an uncommon post-lateral canthoplasty complication. We suggested that surgeons who perform this kind of surgery should ask about epiphora and look for conjunctival sac fistula at follow-up assessment.

## Background

Lateral canthoplasty is a surgical method to beautify eyes by lengthening and enlarging the lateral angle of eyes. It is widely accepted in recent years. However, there are different opinions among people. Some persons regard it as a routine surgery without further consideration while others are neutral due to the related complications [1]. Postoperative complications of lateral canthoplasty include scar, poor alignment of the outer canthus and conjunctival exposure, etc. [2]. Someone reported that lacrimal fistula occurred after upper eyelid reconstruction and lateral canthotomy [3]. But the conjunctival fistula after canthoplasty has not been reported yet. We present a case of conjunctival sac fistula after the lateral canthoplasty.

## Case presentation

**Fig. 1:**
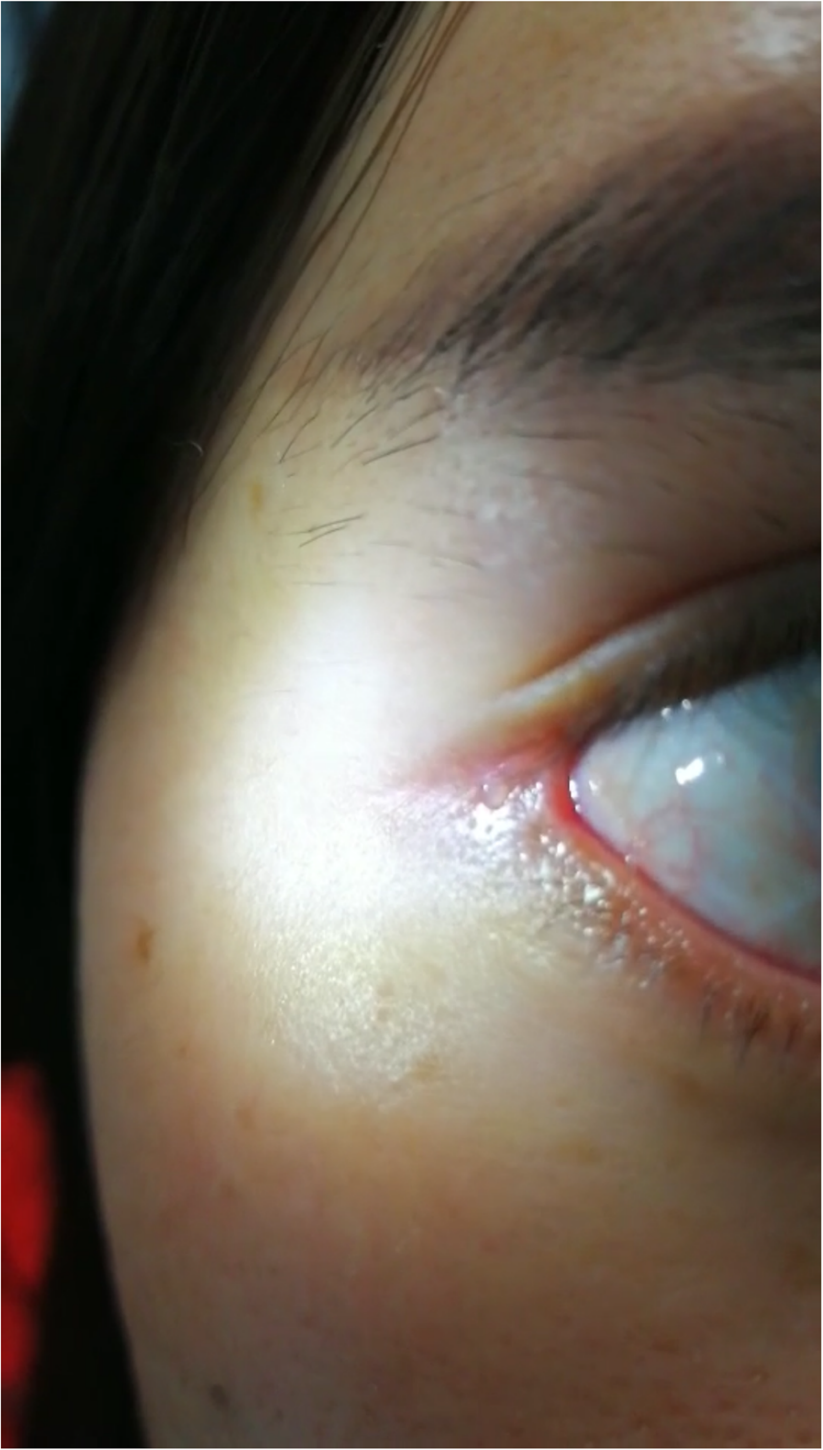
A skin fistula with transparent tears was seen 2 mm lateral to the outer canthus of right eye

**Fig. 2:**
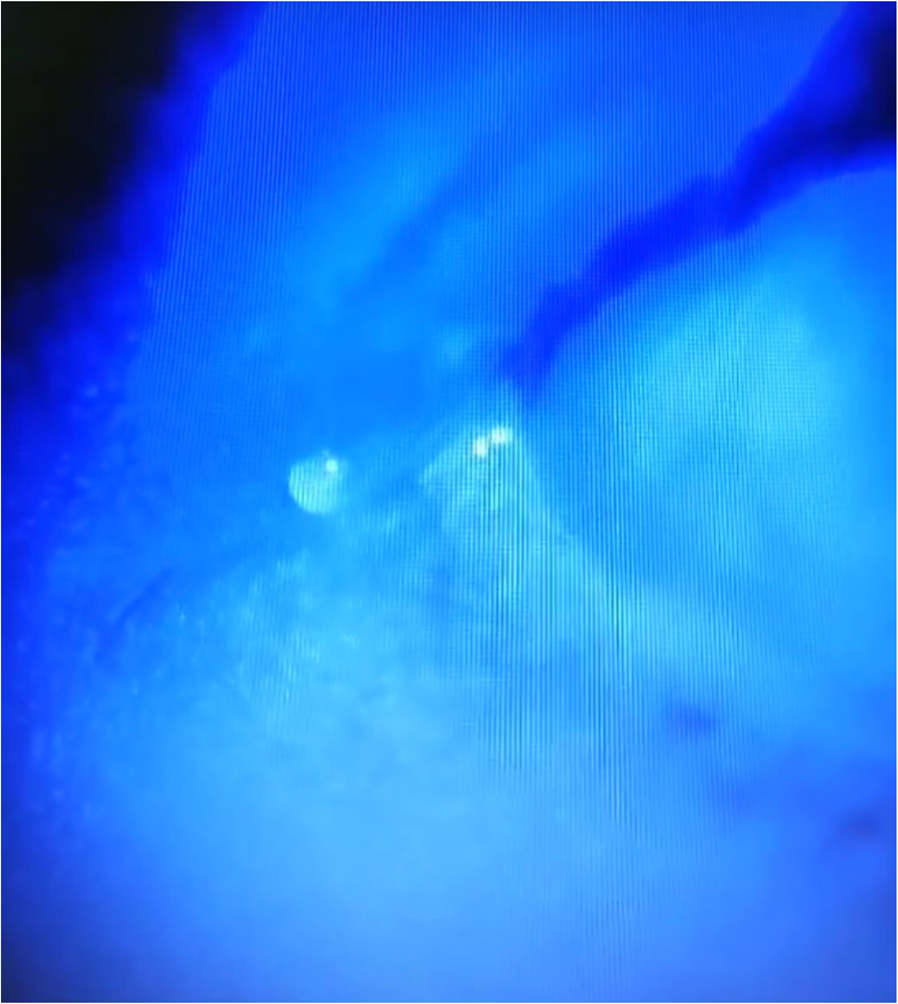
The liquid containing fluorescein came out from the fistula after dropping fluorescein sodium eye drops in conjunctival sac of right eye (Fig. 2)

**Fig. 3:**
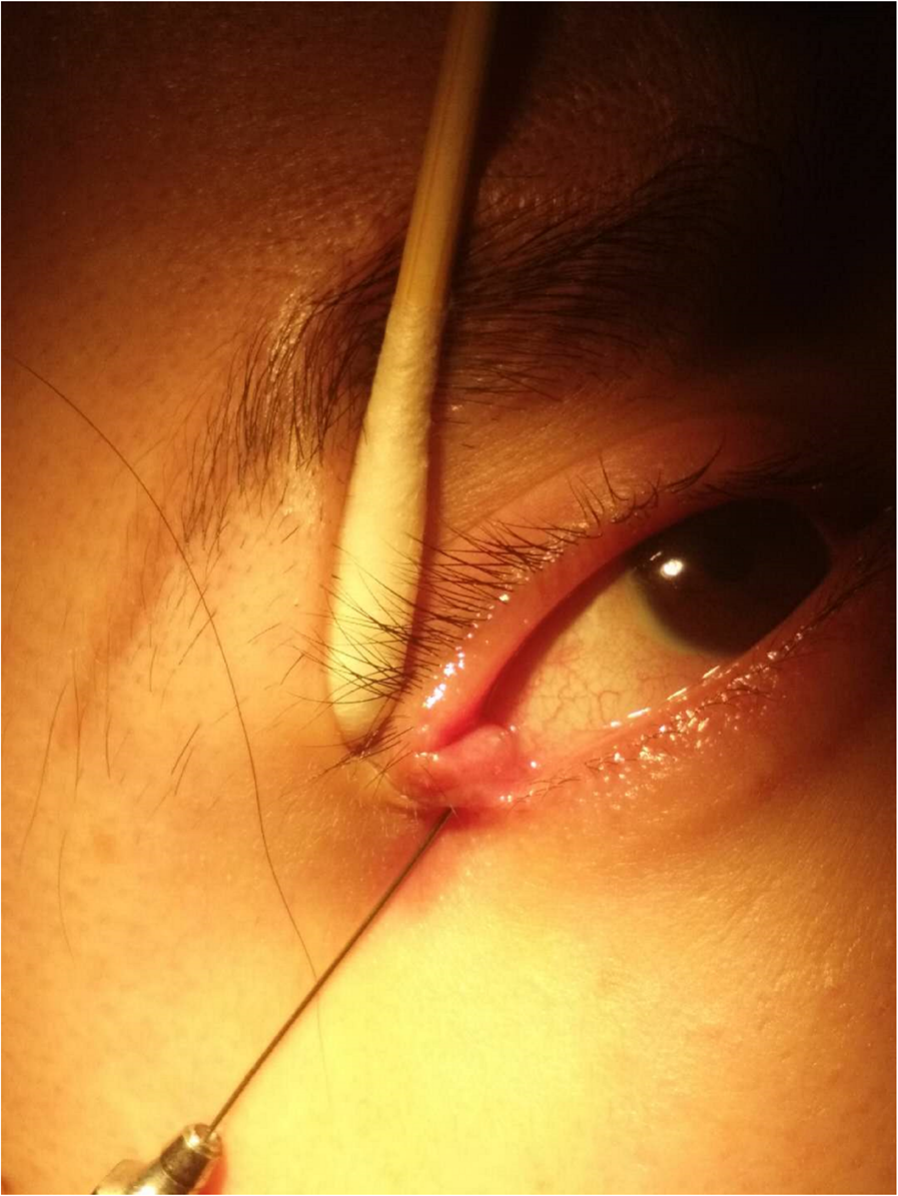
The No. 7 lacrimal duct probe was visible under the temporal conjunctiva and the fistula was about 4 mm in length

A 24-year-old woman complained tears in right eye for 8 months after lateral canthoplasty. She revealed that she underwent the surgery of shortening of levator palpebrae superioris on her right eye and double-eyelid blepharoplasty because of the congenital ptosis of the right eye 4 years ago. She did not feel any discomfort after the surgery. Then she underwent bilateral canthoplasty and epicanthoplasty in another hospital 8 months ago. She presented to our copthalmic clinic for persistent tearing on her right eye. On examination, the best corrected vision was 1.0 in both eyes and the the intraocular pressure was normal. The eyelids of both eyes were symmetrical without malformation. The shin scar was obvious at inner and outer canthus. The lacrimal spots were in the right position and the lacrimal passages were unobstructed. Nevertheless, a skin fistula with transparent tears was seen 2 mm lateral to the outer canthus of right eye (Fig. 1). On further examination, the liquid containing fluorescein came out from the fistula after dropping fluorescein sodium eye drops in conjunctival sac of right eye (Fig. 2). When we try to explore the fistula, the No. 7 lacrimal duct probe was visible under the temporal conjunctiva and the fistula was about 4 mm in length (Fig. 3).

The patient was mainly diagnosed with right lateral canthus-conjunctival sac fistula. The probe was inserted into the fistula and the fistula was excised under local anesthesia. During surgery, we confirmed that the fistula was connected to the temporal conjunctival sac. Besides, the lateral canthal tendon could be observed during exploring. We used the 6–0 absorbable suture (polyglactin 910) to suture the subcutaneous tissue and the 6–0 beauty suture (lingqiao) was used to suture the skin incision. The suture of shin incision was removed 10 days after the surgery. The patient did not tear abnormally after observation 3 months later and the incision healed well. We had obtained the written informed consent from the patient to report the case and adhered to the ethical principles outlined in the Declaration of Helsinki as amended in 2013.

## Discussion and conclusions

Lateral canthoplasty is a method to lengthen the length of the palpable fissure so as to achieve the goal of beautifying eye appearance and it is popular at present. However, some scholars are still cautious about the surgery because the lateral canthus operation may lead to reversible or irreversible results, even for physiological disfunctions.

Mohsen et al. reported that lacrimal fistula occurred in a 25-year-old female patient after surgery of upper eyelid blepharoplasty and shortening of levator palpebrae superioris muscle, who was diagnosed with bilateral ptosis and blepharochalasis. Lacrimal gland restoration and fistula resection were performed and the patient was cured [4]. Ye et al. presented a 34-year-old female with a lacrimal fistula in right eye after upper eyelid reconstruction who underwent lateral canthotomy 4 years ago. Similarly, the patient was cured after lacrimal gland restoration and fistula resection [3]. The common point of the above two cases was the occurrence of lacrimal discharge after upper eyelid plastic surgery and it was associated with the formation of lacrimal gland fistula. Tears are secreted by lacrimal glands and then discharge into the conjunctival sac through the lacrimal duct in the superior fornix. It has been reported that separation of the temporal conjunctival fornix during lateral canthoplasty may damage the lacrimal gland and cause the lacrimal gland dysfunction or tearing [1, 5, 6]. In our case, although the upper eyelid plasty was performed first, the conjunctival sac fistula was formed after lateral canthoplasty. It has not been reported yet.

The formation of canthus-conjunctival sac fistula is considered as the result of growth of eyelid conjunctival membrane epithelial cells in incision. The conjunctival tissue was sutured together with the skin tissue accidentally during surgery. Then a fistula connecting the skin and conjunctival sac is formed because of the proliferation of conjunctival epithelial cells. Lacrimal gland fistula may result from the injury of lacrimal gland ligament and the prolapsed lacrimal gland tissue is sutured with skin. The methods to distinguish the lacrimal gland fistula from canthus-conjunctival fistula have not been reported yet. We believe that the following methods can be useful: 1. If the liquid containing fluorescein comes out from the fistula after using the fluorescein sodium eye drops in conjunctival sac, it can be diagnosed with canthus-conjunctival fistula. 2. If the probe is visible under the conjunctiva when exploring the fistula after using the No. 7 lacrimal duct probe, it is diagnosed with canthus-conjunctival fistula. 3. To do histopathologic examination of fistula resection tissue to find out whether it has the lacrimal gland component or not.

This case report illustrates an uncommon post - lateral canthoplasty complication. We suggested that surgeons who perform this kind of surgery should ask about epiphora and look for conjunctival sac fistula at follow-up assessment.

## Data Availability

Not applicable.
